# An In-Situ Tester for Extracting Piezoresistive Coefficients

**DOI:** 10.3390/mi14040885

**Published:** 2023-04-20

**Authors:** Fengyang Li, Runze Yu, Dacheng Zhang

**Affiliations:** School of Integrated Circuits, Peking University, Beijing 100871, China; lifengyang@pku.edu.cn (F.L.); yrzdx@pku.edu.cn (R.Y.)

**Keywords:** MEMS, parameters extraction, effective stiffness, piezoresistive coefficient

## Abstract

In this study, an electrostatic force-driven on-chip tester consisting of a mass with four guided cantilever beams was employed to extract the process-related bending stiffness and piezoresistive coefficient in-situ for the first time. The tester was manufactured using the standard bulk silicon piezoresistance process of Peking University, and was tested on-chip without additional handling. In order to reduce the deviation from process effects, the process-related bending stiffness was first extracted as an intermediate value, namely, 3590.74 N/m, which is 1.66% lower than the theoretical value. Then, the value was used to extract the piezoresistive coefficient using a finite element method (FEM) simulation. The extracted piezoresistive coefficient was 9.851 × 10^−10^ Pa^−1^, which essentially matched the average piezoresistive coefficient of the computational model based on the doping profile we first proposed. Compared with traditional extraction methods, such as the four-point bending method, this test method is on-chip, achieving automatic loading and precise control of the driving force, so it has high reliability and repeatability. Because the tester is manufactured together with the MEMS device, it has the potential to be used for process quality evaluation and monitoring on MEMS sensor production lines.

## 1. Introduction

Since Smith [1] discovered the piezoresistive effect of silicon and germanium in 1954, it has been widely used in MEMS as the most common sensing principle. The piezoresistive coefficient is the most important physical parameter for the design of all piezoresistive devices, which affects the sensitivity and temperature drift. It depends on factors such as the crystal orientation, doping type, doping concentration, and temperature. Therefore, it is related to the manufacturing process. Additionally, residual stress brought about by the process will also affect the piezoresistive coefficient of the silicon wafer [2]. For a long time, researchers have studied the piezoresistive coefficient through experimental measurement and theoretical calculation. In the early days, Smith [1], Tufte and Stelzer [3], and others cut samples from single crystals and applied tensile tests to them. With the development of semiconductor manufacturing, piezoresistors could be integrated with bulk silicon using diffusion techniques. Matsuda et al. [4] hung weight on a silicon cantilever beam with doped piezoresistors. Recent experiments usually applied force by four-point bending to the piezoresistors [5,6,7]. This method can apply uniform stress without clamping the sample. Jaeger et al. [8], Richter et al. [9], and others also developed test chips for measuring piezoresistive coefficients. Among the above methods, some require special sample preparation, some require external drive instrument alignment, and some require producing test chips. With the development of the large-scale industrialization of MEMS, monitoring and in-situ extraction of the process results have become important for designers, but the methods mentioned above are not applicable to this goal. Thanks to advanced micromachining capabilities, the use of an on-chip drive can minimize alignment error when force is applied. Furthermore, the on-chip extraction we present does not require special test structures or specialized test fixtures, and can in-situ and in real-time reflect the piezoresistive coefficient under the used process, which can provide a reliable reference for MEMS design. Although piezoresistive devices can now be calibrated for sensitivity, nonlinearity, and output drift through the on-chip ASIC [10], the cost and accuracy of calibration are still limited by the performance of the MEMS die. By directly measuring the piezoresistive coefficient on-chip, the distribution of the piezoresistive coefficient on the wafer can be obtained, which is also important to guide the fine-tuning and consistency of the process parameters of the bulk silicon piezoresistance process and predict the sensitivity of piezoresistive devices more precisely [11].

Our tester has the following characteristics: (1) it is compatible with standard MEMS processes and can be manufactured together with the MEMS device; (2) it only requires a small area on the wafer as a detection unit; and (3) it contains a simple drive structure and principle to accurately reflect the target parameter without being affected by other parameters. The electrostatic drive has a fast response, a simple structure, and precise control. Therefore, electrostatic pull-in measurement is an effective choice. This method was first proposed by Petersen et al. [12], and later developed by Osterberg and Senturia [13]. The mainstream silicon-based piezoresistive technologies use boron-doped p-type piezoresistors in MEMS sensors. In p-type silicon, the piezoresistive effect is mainly described by the shear coefficient *π*_44_, which is also the most often considered piezoresistive coefficient of MEMS piezoresistive sensors. In this paper, we focus on *π*_44_ and present the tester structure and test method.

The extraction of the piezoresistive coefficient through beam bending requires stress distribution on the beam, and the bending stiffness is a key parameter. This is also an important mechanical parameter related to process quality in MEMS devices. The ion implantation [14] and heat treatment [15] will have a certain influence on the elastic stiffness of silicon. At the same time, different production lines will cause different types of surface damage, which also affects the stiffness of the beam. To obtain the actual stress distribution on the beam, this paper first extracted the bending stiffness of the beam, containing the process influence. This value was used for the FEM simulation of the stress in the piezoresistive regions.

In this paper, based on the above considerations, we present a tester consisting of an electrode on a glass substrate and a mass supported by four guided cantilever beams (FGCBs). Compared with the reported pull-in structure, the FGCBs with the mass at the center have greater rigidity and stability and provide a reliable response [16]. In Section 2, we first introduce the structure of the tester and its advantages, show the details of the tester design, and introduce two key parameter extraction methods. We also discuss the correlation between the piezoresistance process parameters and the piezoresistive coefficient, as well as the impact of the doping profile on the piezoresistive coefficient. In Section 3, we discuss the influencing factors behind the uncertainty. Our standard bulk silicon piezoresistive process, which was used to manufacture a series of piezoresistive sensors, is presented in Section 4. Finally, the manufactured testers were tested at room temperature (300 K) and compared with the theoretical model, which successfully extracted the effective stiffness and piezoresistive coefficient under this process. Additionally, the results confirmed the influence of the process on the mechanical properties.

## 2. Theoretical Model and Design Concept

### 2.1. Structure of FGCBs

**Figure 1:**
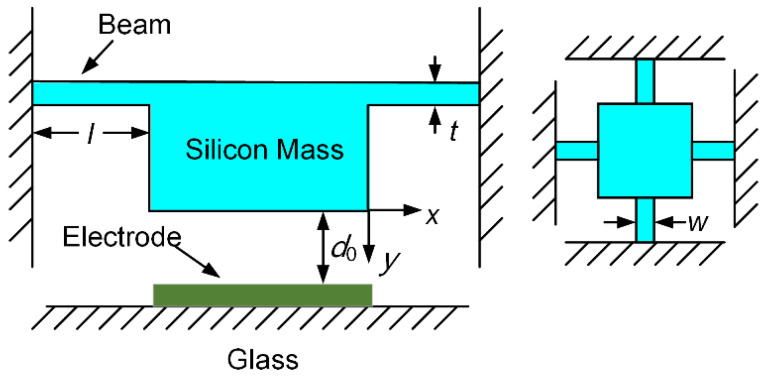
Schematic diagram of the tester: side view and top view. The key dimensions are marked in the picture.

**Figure 2:**
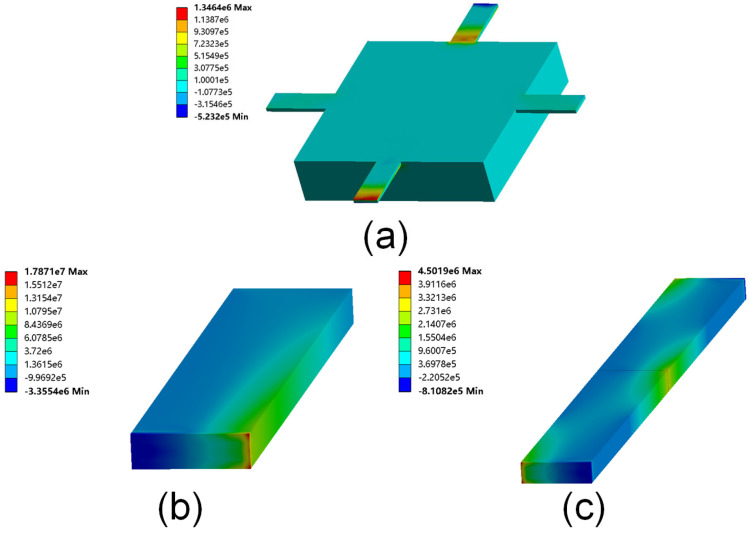
The first principal stress cloud diagram when applying a lateral force (surface load) to the (**a**) FGCB structure (mass underside), (**b**) cantilever beam (free end), and (**c**) fixed-fixed beam (middle section) of the same geometric sizes.

The schematic diagram of the tester is shown in Figure 1. The tester consists of an electrode on a glass substrate and a mass supported by four guided cantilever beams (FGCBs), and a piezoresistive Wheatstone bridge is integrated on the beams. The movable mass can be driven by the driving voltage between the mass and the electrode. In the previous research on the pull-in phenomenon, fixed–fixed beams [17] and cantilever beams [18] were usually used. These models are hard to solve analytically due to changes in the plate angle and electric field distribution. FGCBs have a simpler and more accurate mechanical model because the bottom of the mass is always parallel to the electrode on the glass substrate. Moreover, when applying a lateral force (10% of the restoring force during pull-in) to a cantilever beam, a fixed–fixed beam, and our structure with the same feature sizes, the first principal stress on the FCGBs is the smallest, as shown in Figure 2. Therefore, FGCBs can effectively resist lateral perturbance through accidental factors near the critical point. Since the stiffness of the glass substrate is high enough and the four beams have good elasticity, it is believed that the glass is nondeformable, and that the change in the gap is only caused by mass movement. Further, it can be approximated that there is no deformation in the mass area because its thickness is much larger than that of the beams. Due to the symmetry of the structure, the movement of the mass is translation along the y direction.

**Figure 3:**
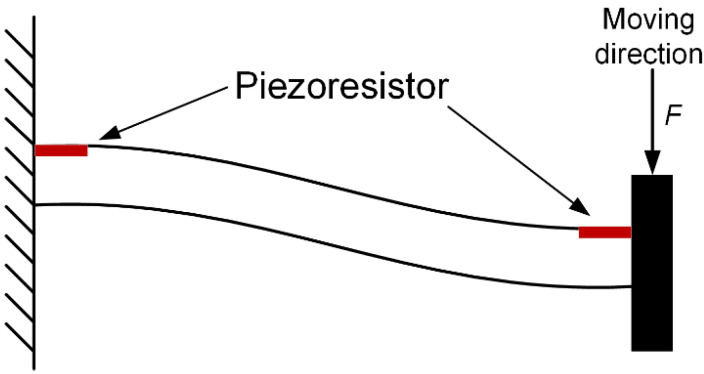
The side view of a guided cantilever beam (fixed end and guided end) and a beam that has two piezoresistors on it.

In our structure, the beam is a guided cantilever beam (fixed-guided beam), as shown in Figure 3. When a force *F* is applied, the displacement *y* of the guided end can be expressed as

(1) $$y=\frac{Fl^{3}}{12EI}$$

Therefore, the bending stiffness *K* is given by

(2) $$K=\frac{Force}{Deflection}=\frac{12EI}{l^{3}}=\frac{Ewt^{3}}{l^{3}}$$

where *E* is Young’s modulus, and *I* is the moment of inertia of the beam. Additionally, *l*, *w*, and *t* are the length, width, and height of the beam, respectively. The designed structure sizes of the tester and measured value are shown in Table 1.

### 2.2. Pull-In Model

The FGCBs structure is equivalent to four parallel springs. When voltage is applied to the electrode on the glass and the mass, the resultant force on the mass is

(3) $$F=-\frac{A\varepsilon_{0}V_{s}{}^{2}}{2{\left(d_{0}-y\right)}^{2}}-4K_{eff}y$$

where *A* is the base area of the mass (the same as the electrode); *V_s_* the driving voltage; d_0_ is the capacitor gap at *V_s_* = 0; *K_eff_* is the effective stiffness of the beam; *y* is the displacement of the mass; and *ε*_0_ is the dielectric constant in vacuum. The minimum electrostatic voltage at which pull-in occurs (*V_pmin_*) is the critical voltage at *y* = *d*_0_/3. At this moment, the effective stiffness can be obtained:

(4) $$K_{eff}=\frac{27A\varepsilon_{0}V_{pmin}^{2}}{32d_{0}^{3}}$$

The expected pull-in voltage of the tester will fall within the range of 5–100 V, because this is the typical voltage range of MEMS electrical experiments. The structure size of the tester listed in Table 1 also includes this consideration.

### 2.3. Piezoresistive Coefficient Measuring Circuit

The piezoresistors are along the <110> direction on a p-type (100) silicon wafer. The piezoresistive coefficients *π*_11_ and *π*_12_ of the p-type lightly doped piezoresistor are much smaller than the shear piezoresistive coefficient *π*_44_ [1]. Therefore, the longitudinal and transverse piezoresistive coefficients of the piezoresistors are as shown in Equation (5) and the relative change of the resistance are as shown in Equation (6), whose relative error is less than 5%.

(5) $$\begin{array}{l} \pi_{l}=\frac{\pi_{11}+\pi_{12}+\pi_{44}}{2}\approx \frac{\pi_{44}}{2}\\ \pi_{t}=\frac{\pi_{11}+\pi_{12}-\pi_{44}}{2}\approx -\frac{\pi_{44}}{2} \end{array}$$

(6) $$\frac{\Delta R}{R}=\pi_{l}\sigma_{l}+\pi_{t}\sigma_{t}=\frac{\pi_{44}}{2}(\sigma_{l}-\sigma_{t})$$

**Figure 4:**
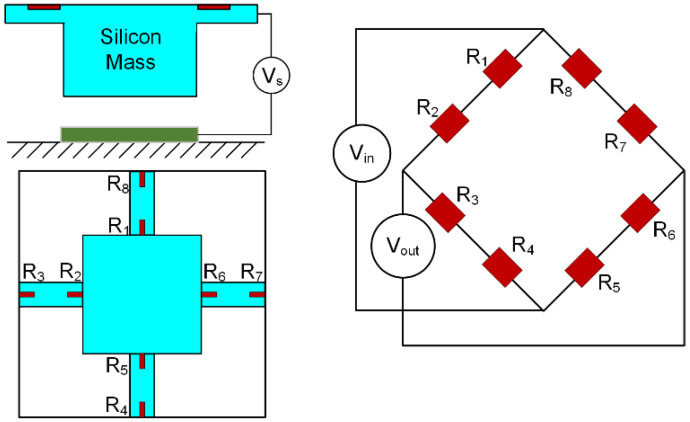
Schematic diagram of piezoresistors’ distribution and the electrical connection.

In this experiment, because the maximum tensile stress on the beam is generated at the fixed end and the maximum compressive stress is generated at the guided end when the mass moves, two piezoresistors were designed both at the guided and fixed ends of each beam, as shown in Figure 3. The stress is symmetrical at both ends [19]. The eight piezoresistors are connected to form a Wheatstone bridge, and the circuit connection of the whole system is shown in Figure 4. The initial resistance values of the eight resistors are equal for the same manufacturing process. The typical value for the resistors is 1.4 kΩ. During the deformation, the change in *R*_1_, *R*_2_, *R*_5_, and R_6_ is Δ*R*_1_, and the change in *R*_3_, *R*_4_, *R*_7_, and *R*_8_ is Δ*R*_2_. For actual results, it is difficult to ensure that the stresses in the tensile zone and compression zone are exactly the same. Combined with the expression of the bridge, the output voltage *V_out_* of the tester is

(7) $$V_{out}=V_{in}\frac{\Delta R_{2}-\Delta R_{1}}{2R_{0}+\Delta R_{2}+\Delta R_{1}}=V_{in}\frac{\sigma_{lt2}-\sigma_{lt1}}{4/\pi_{44}+\sigma_{lt2}+\sigma_{lt1}}$$

where *σ*_*lt*1_ is the average of the difference between the transverse and longitudinal stresses in the compression region; *σ*_*lt*2_ is the average of the difference between the transverse and longitudinal stresses in the tension region; and *V_in_* is the bridge input voltage, which is a typical DC voltage of 5 V [20]. In order to obtain accurate results of *π*_44_, the piezoresistive area is meshed to calculate the average stress through FEM simulation.

### 2.4. Piezoresistive Coefficient Based on Doping Concentration Distribution

Process parameters, such as the dose and energy of the dopant, temperature and time of annealing, and thermal oxidation conditions, will affect the distribution of the doping concentration; different doping concentrations determine different piezoresistive coefficients. In order to study the correlation between the process parameters and piezoresistive coefficient, first, the SPROCESS module of the process and the device simulation tool Sentaurus TCAD were used to establish the correlation between the process parameters and the doping concentration distribution. In the following, the calculation method of the piezoresistive coefficient based on the doping concentration distribution is discussed.

The piezoresistive coefficient is closely related to the doping concentration (*N*) and temperature (*T*). Kanda [21] presented a theoretical model for piezoresistive coefficients related to the doping concentration and temperature. He expressed the effect of the doping concentration and temperature as the piezoresistive factor *P*.

(8) $$\pi (N,T)=P(N,T)\pi_{\mathrm{ref}}$$

where π_ref_ is the piezoresistive coefficient at 300 K and a low doping concentration. The π_ref_ of *π*_44_ in p-type silicon is 138.1 × 10^−11^ Pa^−1^. The research and revision of the basic theoretical model of piezoresistive coefficients had not made any breakthroughs until recent years. The latest model of *P* is the modification given by Joseph et al. [22] based on the Richter model [11], which was used in our work:

(9) $$P(N,T)=\frac{T_{n}^{-0.95}}{1+{\left(N/4.9\times 10^{19}\right)}^{0.39}T_{n}^{-1.35}+{\left(N/2.6\times 10^{20}\right)}^{0.94}T_{n}^{-4.55}}$$

where *T_n_* = *T*/300.

**Figure 5:**
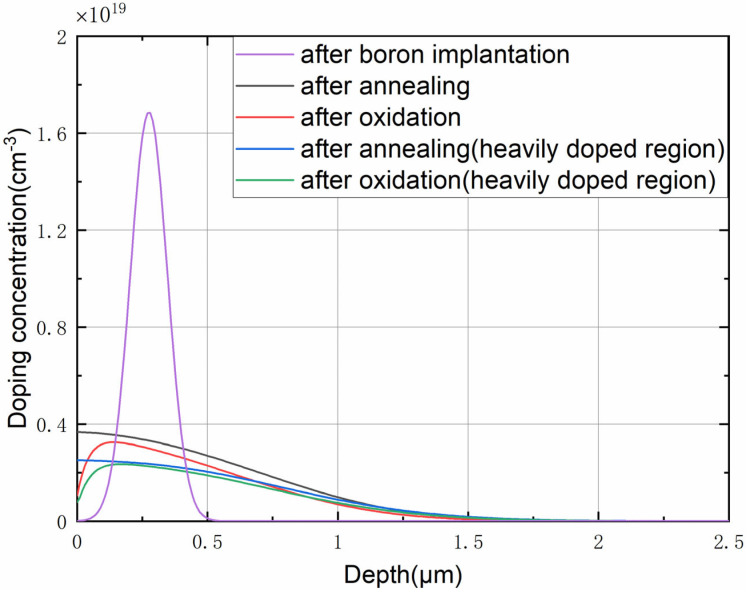
Doping concentration distribution of the piezoresistor based on the standard bulk silicon piezoresistance process flow obtained via SPROCESS simulation.

Figure 5 shows the doping concentration distribution curve *N*(*z*) of the piezoresistor based on the typical bulk silicon piezoresistance process of SPROCESS. The distribution of the doping concentration in depth shows that the piezoresistive effect exhibited is the average result of all thin layers. The layer with high conductance carries most of the total current, so the conductance of the single-crystal silicon needs to be calculated, which is

(10) σ(N,T)=qμ(N,T)N

where *q* is the unit charge (constant), and *μ* is the hole mobility. Additionally, the model proposed by Arora et al. [23] for μ is widely used:

(11) $$\mu (N,T)=54.3T_{n}{}^{-0.57}+\frac{1.36\times 10^{8}T^{-2.23}}{1+0.88NT_{n}{}^{-0.146}/2.35\times 10^{17}T_{n}{}^{2.4}}$$

Combined with the doping profile *N*(*z*) in depth calculated using SPROCESS, the distribution curve of the conductance *σ*(*z*) and piezoresistive coefficient *π*(*z*) can be obtained through Equations (8) and (10). The average piezoresistive coefficient is the average result exhibited on the basis of the conductance distribution in the layer. *π* × *σ* reflects the distribution of the conductance change under unit stress in diffused depth, and by dividing the total amount of change in conductance under unit stress by the total conductance, the average piezoresistive coefficient can be obtained [3]:

(12) $$\pi_{ave}=\int_{0}^{H}\sigma (z,T)\times \pi (z,T)dz/\int_{0}^{H}\sigma (z,T)dz$$

where *H* is the depth of the diffused layer.

## 3. Analysis of Measurement Uncertainty

Equations (4) and (7) can be used to extract the effective stiffness of the beam and the piezoresistive coefficient. However, this is obtained under ideal conditions. The process will inevitably bring measurement uncertainty. The main factors leading to uncertainty are the uncertainty of alignment error, line width, and effects of vibration and temperature.

### 3.1. Alignment Error

**Figure 6:**
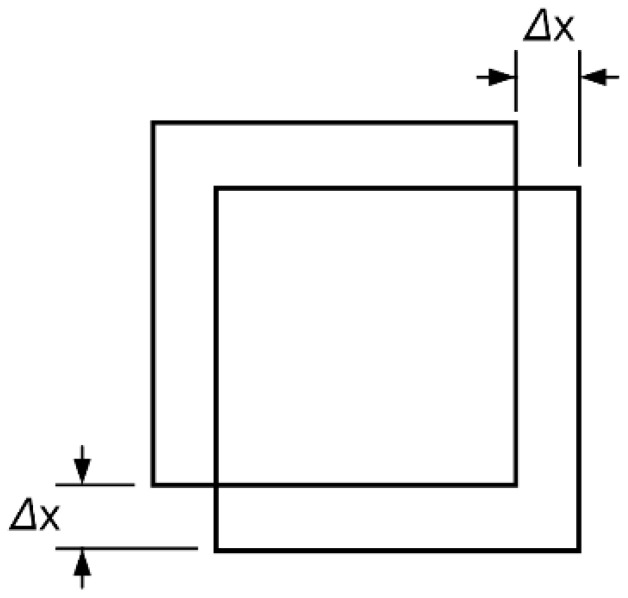
Schematic diagram of the alignment deviation between the bottom surface of the mass and the electrode on the glass.

Bonding alignment brings uncertainty in position. In our technological environment, the maximum alignment error Δ*x* was ±2 μm. The effect was a reduction in the effective area of the capacitor. Considering the maximum offset of bonding alignment shown in Figure 6, the change in the area of the capacitor plate was 1.58 × 10^4^ μm^2^ and the uncertainty was less than 1%.

### 3.2. Line Width

The deviation of the etching will cause the loss of the line width, which is manifested in the lateral undercutting. This will cause the cross-section of the beam to be approximately an isosceles trapezoid. In our technological environment, the maximum loss of the line width was 1.2 μm. The moment of inertia of the beam is

(13) $$I=\frac{t^{3}(a^{2}+4ab+b^{2})}{36(a+b)}$$

where *a* and *b* are the top and bottom of the trapezoid, respectively. In the worst case, the influence of the line width on stiffness was less than 1%, as calculated using Equations (2) and (13).

### 3.3. Vibration

Near the critical position where pull-in occurs, small vibrations may induce pull-in. Thanks to the extremely small gap relative to the area of the capacitor plate, the squeeze film damping lowers the quality factor *Q* of the resonance. In addition, when we increased the voltage across the capacitor, the step size was 0.2 V with a large time constant. The resolution of the corresponding effective stiffness measurement was 19.95 N/m, approximately 0.55% of the theoretical value. The influence of vibration interference on the test accuracy can be ignored.

### 3.4. Temperature

We extracted the piezoresistive coefficient at room temperature (300 K), but temperature fluctuations during the measurement process may affect the thermal coefficients of resistance. We measured resistance in the environment of a temperature control box. The typical resistance was 1407.19 Ω at 300 K, and the change caused by a temperature change of 1 K was 8.694 Ω. The corresponding stress according to Equation (6) was 9.301 × 10^5^ Pa. The stress in the piezoresistor region during pull-in was 1.2 × 10^8^ Pa from FEM, with a corresponding uncertainty of 7.75%. The temperature can be controlled in the chamber within 0.5 K. Therefore, the uncertainty from thermal effects was 3.88%.Therefore, the total measurement uncertainty was less than 4.17% by calculating the square-root of the sum of squares of the errors in each part mentioned above.

## 4. Fabrication Process

**Figure 7:**
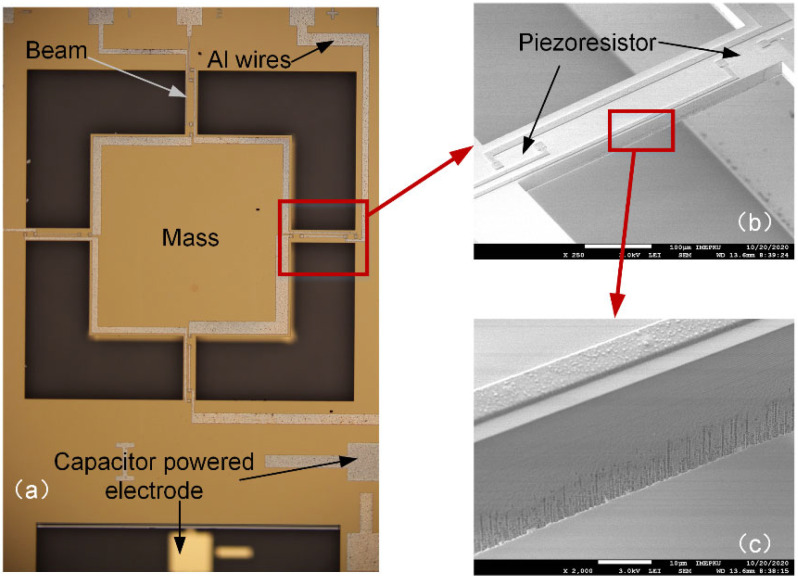
(**a**) Photograph of the tester under an optical microscope. *V_s_* was applied on the capacitor powered electrode. (**b**) SEM image of the beam. Piezoresistors were placed at the ends of the beams. (**c**) Surface damage caused by RIE on the beam sidewall.

**Figure 8:**
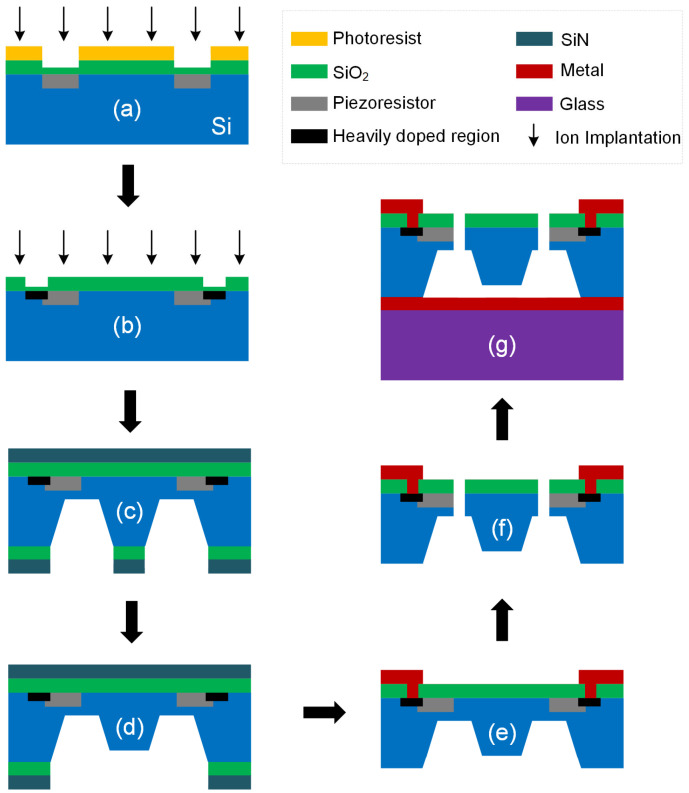
Flow of the fabrication process. (**a**) Light doping to form piezoresistors. (**b**) Heavy doping to form the connection region. (**c**) KOH etching on the back side. (**d**) Simultaneous mass and bulk silicon thinning to form the capacitor gap. (**e**) Metalization to form the circuit. (**f**) RIE etching from the front. (**g**) Si–glass anodic bonding.

The structure of the tester was manufactured following the standard bulk silicon piezoresistive process of Peking University, which possesses the following characteristics: (1) MEMS piezoresistive sensors can be manufactured; (2) suspended beam structures can be manufactured; and (3) the process flow is compatible with mainstream MEMS manufacturing processes, and it is easy to extend to large-scale production lines. First, we used the ion implantation process to create the piezoresistive area and heavily doped area. Then, the KOH etching process was performed twice to form the mass and capacitor gap. Additionally, the circuit was formed via Al (aluminum) sputtering and the lift-off process. The last step was deep reactive ion etching to release the silicon structure, thereby achieving the manufacture of the MEMS suspension structure, FGCBs. The entire structure was bonded to the glass. Photographs of the fabricated tester are shown in Figure 7. The process flow is shown in Figure 8.

Our tester was manufactured together with the MEMS device under the same process. The conditions of the standard bulk silicon piezoresistance process of Peking University are as follows: boron ion implantation is performed at a concentration of 3 × 10^14^ cm^−2^ and an implantation energy of 100 keV to form piezoresistors. Then, annealing at 1100 °C/120 min and oxidation at 1000 °C/37 min are carried out. The heavily doped connection region is prepared via boron diffusion to realize an effective ohmic contact. These are used to calculate the theoretical piezoresistive coefficient in Section 5.2.

## 5. Experimental Results and Discussion

### 5.1. Effective Stiffness

**Figure 9:**
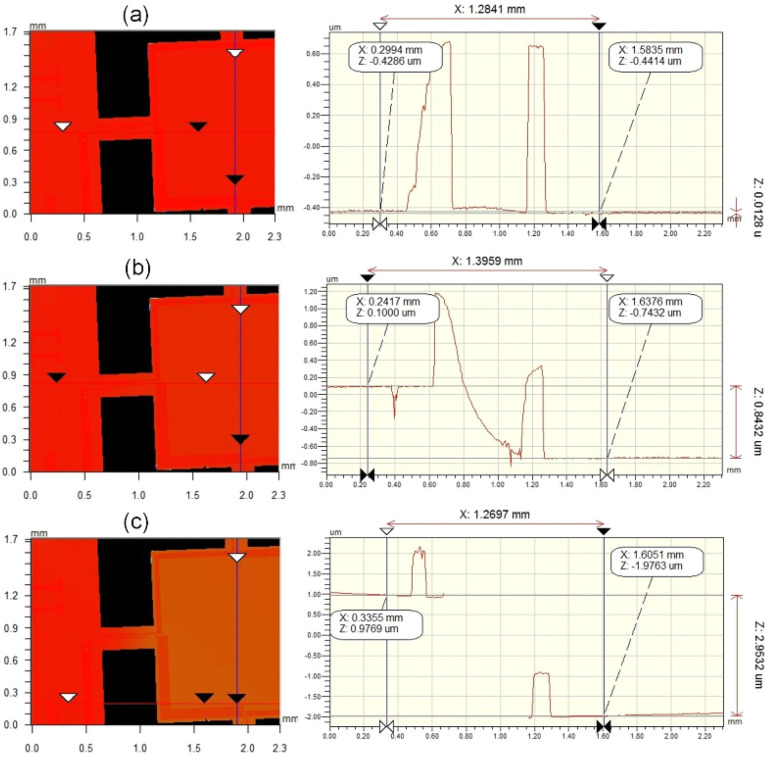
Pull-in process observed by optical profiler. (**a**) When the driving voltage was 0 V, the step height was 12.8 nm, and it was considered that the mass had no downward displacement. (**b**) When the driving voltage was 71.0 V, the step height increased to 843.2 nm. (**c**) When the driving voltage was 71.2 V, pull-in occurred, and the step height was 2.95 μm. Therefore, the minimum value of the pull-in voltage *V_pmin_* of this structure was 71.2 V (the accuracy was within 0.2 V).

**Figure 10:**
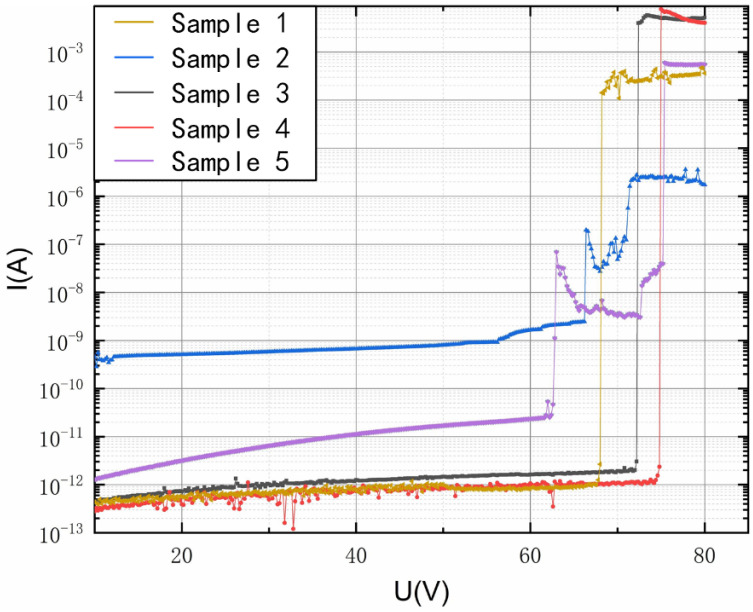
Current curves of detection circuits from several testers. The current changes suddenly at the pull-in point.

We used the semiconductor parameter analyzer HP4156B to apply a voltage *V_s_* between the electrode on the glass and the mass. Additionally, we observed pull-in from the electric current change combined with the signal of an optical profiler. The use of the optical profiler was not necessary, and it was only used to display the process of pull-in in this paper. To ensure the quasi-static state of the system, this voltage increased from zero slowly. The downward translation distance of the silicon mass could be accurately measured by the profiler. Because the gap is extremely small, particles in the air may cause air breakdown, thus causing fluctuations or even false peaks in the current curve. The profiler can calibrate the test. When pull-in occurs, the current passing through *V_s_* will change suddenly. The profiler can accurately determine the pull-in point, avoiding interference if a possible noise peak occurs. In addition, the gap could be accurately measured with the profiler image during pull-in, as shown in Figure 9(c). To fully reflect the process quality of the silicon wafer, the bonded wafer was partitioned into five zones and multiple measurements (five) were carried out in each zone to ensure the reliability of the test results. Figure 9 shows the process of the movement of the mass. The results of the detection circuit of different test regions are shown in Figure 10. The effective stiffness can be obtained by substituting the pull-in voltage into Equation (4). Five sets of data are shown in Table 2.

Combined with the tester sizes, the average of the effective stiffness *K_eff_* was 3590.74 N/m, and the “effective Young modulus” was calculated to be 166.2 GPa using Equation (2). The maximum deviation of the measurement was 392.14 N/m. The deviation percentage was 10.92%. The test demonstrated good repeatability. The results show that the experimental value was 1.66% lower than the 169 GPa of the macroscopic bulk silicon material [24]. Possible causes include the heat treatment and surface defects. The results reflect the influence of the process on the mechanical properties and, at the same time, present the process uniformity on the wafer. The effective stiffness of the beam was extracted, which is of great significance for the design of high-reliability MEMS devices.

### 5.2. Piezoresistive Coefficient

**Figure 11:**
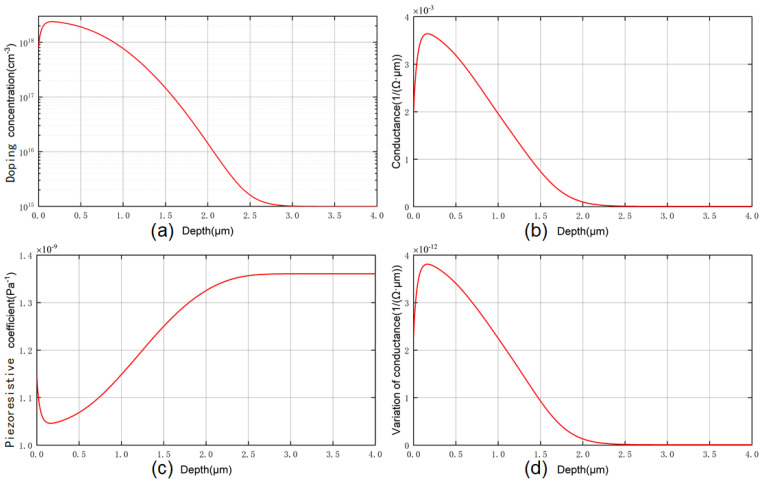
(**a**) Doping concentration distribution under our process obtained via SPROCESS simulation. The distribution of the piezoresistive electrical parameters in depth is also shown: (**b**) conductance; (**c**) piezoresistive coefficient; (**d**) variation in conductance under unit stress.

The SPROCESS module of the process and the device simulation tool Sentaurus TCAD were used to obtain the doping concentration curve under the process conditions presented in Section 4, as shown in Figure 11(a). Figure 11(d) shows the distribution curve of *π* × *σ* in depth. We employed numerical integration for the above curve to obtain the total amount of change in conductance under unit stress, and the average piezoresistive coefficient *π*_44_ calculated using Equation (12) was 1.097 × 10^−9^ Pa^−1^.

**Figure 12:**
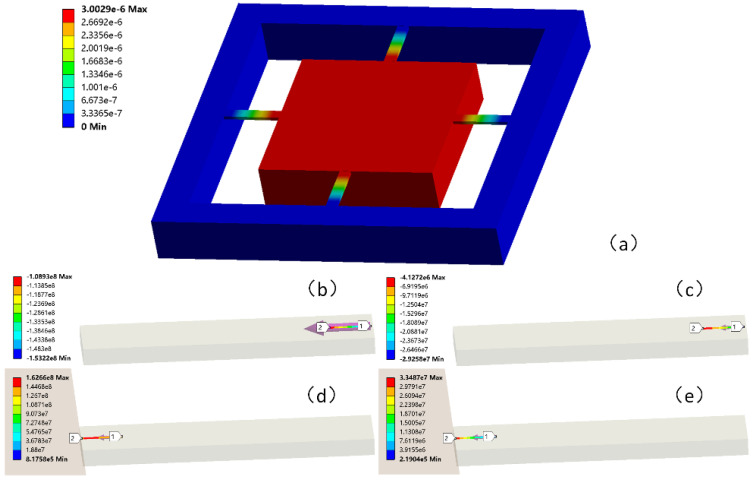
FEM simulation results during pull-in. (**a**) Total deformation of the tester structure. (**b**,**c**) *σ*_l_ and *σ*_t_ in the piezoresistor compression region. (**d**,**e**) *σ*_l_ and *σ*_t_ in the piezoresistor tension region.

For simplicity, we can increase the driving voltage to the pull-in voltage or higher, so that the mass is attracted to the glass substrate. At this time, there should be a very stable output. Since the stress at each point on the piezoresistor is different, we employed numerical integration for the variation in the resistance value to obtain an accurate solution. For the structure at the time of pull-in, the stress distribution in the piezoresistive region was obtained via FEM simulation, as shown in Figure 12. The stress in the piezoresistive zones conformed to a linear distribution. The average of the difference between the transverse and longitudinal stresses in the compression region was −1.18 × 10^8^ Pa, and the average of the difference between the transverse and longitudinal stresses in the tension region was 1.20 × 10^8^ Pa. Additionally, the *V_out_* of the Wheatstone bridge was 292.89 mV at this time. *π*_44_ was calculated using Equation (7) to be 9.845 × 10^−10^ Pa^−1^. It can be seen from the FEM analysis result that the average transverse and longitudinal stress differences between the tension and compression regions were roughly equal in value.

In different partitions of the wafer, we calculated the piezoresistive coefficient by using the output voltage at pull-in, combined with FEM. The results are shown in Table 3. The average of the piezoresistive coefficient *π*_44_ was 9.851 × 10^−10^ Pa^−1^. The maximum deviation of the measurement was 0.723 × 10^−10^ Pa^−1^. The deviation percentage was 7.34%. The experimental results showed a 10.2% deviation from the model. This result is considered reliable because the Arora and Joseph models themselves have an error in the experimental measurement value. Arora pointed out that the relative error between his formula and the experimental value was within ±13% [23].

## 6. Conclusions

In this work, an electrostatic-force-driven tester was introduced to achieve the in-situ extraction of the effective stiffness and piezoresistive coefficient. Compared with the previously reported extraction method, this tester has unique advantages in extracting process-related parameters and is suitable for MEMS sensor production lines. First, we extracted the bending stiffness of the beam structure, recorded the pull-in curves of different zones on the wafer, and carried out calibration with a profiler. The experimental results showed that the influence of the manufacturing process could cause the “effective Young modulus” of the silicon beam to deviate from its classical value by 1.66%. The experimental deviation percentage was 10.92%. Then, we used this value to perform FEM simulation, and obtained a more accurate stress distribution, which reduced the error from the process influence. Combined with the bridge output, the piezoresistive coefficient was calculated to be 9.851 × 10^−10^ Pa^−1^. The experimental deviation percentage was 7.34%. We discussed the correlation between the piezoresistance process parameters and the piezoresistive coefficient, and calculated the average piezoresistive coefficient considering the doping profile. The piezoresistive coefficient essentially matched that of the existing model.

However, our process-related evaluation is incomplete because FEM simulation does not consider streaks and surface defects. Additionally, the experiment did not fully study the possible effects of residual stress. Nonetheless, we provide a new idea for the measurement of the piezoresistive coefficient: transfer the measurement on-chip and contribute to studying the process dependence of the piezoresistive coefficient. This test has high reliability and repeatability. The presented tester provides a technical path for evaluating the process-related bending stiffness and piezoresistive coefficient, with potential to be used for lot monitoring on MEMS sensor production lines.

## Figures and Tables

**Table 1:** Designed geometric parameters (shown in Figure 1). *L* and *W* are the length and width of the piezoresistor, respectively.

Design Parameter	Measured Value from SEM	Difference (%)
*l* = 500 μm	*l* = 501.3 μm	0.26
*w* = 100 μm	*w* = 100.5 μm	0.5
*t* = 30 μm	*t* = 30.8 μm	2.7
*A* = 2.5 × 10^6^ μm^2^	*A* = 2.5 × 10^6^ μm^2^	-
*d*_0_ = 3 μm	*d*_0_ = 2.95 μm	1.6
*L* = 70 μm	-	-
*W* = 10 μm	-	-

**Table 2:** Effective stiffness data for several testers.

Sample Number	Pull-In Voltage (V)	Effective Stiffness (N/m)	Effective Young’s Modulus (GPa)
1	68.0	3198.6	147.9
2	71.0	3487.0	161.2
3	71.2	3506.7	162.1
4	74.6	3849.6	178.0
5	75.2	3911.8	180.8

**Table 3:** Shear piezoresistive coefficient data for several testers.

Sample Number	Pull-In Voltage (V)	Shear Piezoresistive Coefficient (Pa^−1^)
1	68.0	10.556 × 10^−10^
2	71.0	10.170 × 10^−10^
3	71.2	9.845 × 10^−10^
4	74.6	9.554 × 10^−10^
5	75.2	9.128 × 10^−10^
